# Enhancing Sulfidization and Flotation of Smithsonite Using Eco-Friendly Triethanolamine: Insights from Experimental and Simulation Studies

**DOI:** 10.3390/molecules29143433

**Published:** 2024-07-22

**Authors:** Song Zhang, Guanyu Liang, Yongjun Xian, Shuming Wen

**Affiliations:** 1 State Key Laboratory of Complex Nonferrous Metal Resources Clean Utilization, Kunming University of Science and Technology, Kunming 650093, China; zskmust@126.com (S.Z.); shmwen@126.com (S.W.); 2 Faculty of Land Resource Engineering, Kunming University of Science and Technology, Kunming 650093, China

**Keywords:** smithsonite, triethanolamine, surface sulfidization, flotation recovery, molecular dynamics simulation

## Abstract

Triethanolamine (TEA) is a promising eco-friendly alternative to inorganic ammonia for enhancing surface sulfidization and flotation recovery of smithsonite. Micro-flotation experiments revealed an enhancement in smithsonite recovery to 95.21% with TEA modification, comparable to the results obtained using ammonia. The mechanisms behind the ability of TEA to enhance the sulfidization process were investigated through surface analysis and molecular dynamics simulations. TEA modification increased the content of sulfidization products, the proportion of crucial S_2_^2−^ in adsorbed products, and the thickness and size of the sulfidization product layer. The complexation of TEA with Zn sites formed positively charged Zn–TEA complexes that adsorb onto the smithsonite surface. These complexes promoted negatively charged HS^−^ adsorption, creating a multi-layered adsorption structure. Moreover, TEA modification reduced the total energy required for the sulfidization. These findings open up new possibilities for using eco-friendly reagents in mineral processing, highlighting the potential of TEA in green mineral processing practices.

## 1. Introduction

Zinc, as a critical metal, predominantly comes from zinc sulfide minerals [1]. However, the exploitability of these sulfide resources is diminishing as a result of the escalating global demand for zinc. In this context, the high-efficiency utilization of zinc oxide resources has become increasingly significant [2]. The Huoshaoyun lead–zinc deposit in Hotan County, Xinjiang, China, is one of the highest-grade deposits in Asia, with zinc reserves close to 19 million tons. The Jinding deposit in Lanping, Yunnan Province, was once the largest zinc–lead deposit in China, with zinc reserves that exceed 12.84 million tons. In particular, one-third of the zinc in the Jinding deposit consists of zinc oxide minerals, while in the Huoshaoyun deposit, smithsonite is the mineral form of zinc carbonate (ZnCO_3_), and accounts for 85% of the total zinc resources [3,4,5,6,7]. Direct extraction of zinc from ores through pyrometallurgical or hydrometallurgical methods is often technically complex and costly due to the low zinc concentration and the presence of numerous impurities in zinc oxide ores [8]. To address this issue, mining companies commonly use flotation methods to preseparate and enrich oxidized zinc minerals [9,10,11,12,13,14]. However, the strong polarity and high solubility of the smithsonite surfaces make them prone to forming hydrophilic hydration films with water molecules, which hampers the adsorption of collectors, and thus reduces the flotation performance. Therefore, in practice and scientific research, the surface sulfidization treatment of oxidized zinc minerals is essential before using amine or xanthate collectors for flotation. These Zn–S products are formed on mineral surfaces during the sulfidization process, which enhance the collection and facilitate hydrophobic flotation of oxidized zinc minerals [15,16,17]. Consequently, the surface sulfidization of zinc oxide minerals is considered a key factor that affects the efficiency of their flotation process.

In fact, the effectiveness of sulfidization treatment on the surface of smithsonite is limited by the quantity and stability of the sulfidization products, which presents challenges in achieving effective sulfidization [18,19,20,21,22,23]. Modifications with ammonium salts or ammonia [22,24,25,26,27,28] and treatment with heavy metal ions such as lead and copper have been attempted to enhance the sulfidization degree [29,30,31]. Among these, modification with ammonia–ammonium compounds plays a multifaceted role in the promotion for surface sulfidization and flotation of zinc oxide minerals. Ammonia or ammonium salts can form complexes with zinc ions on mineral surfaces, altering surface charge distribution and increasing the reactivity, which facilitates the reaction of sulfidizing reagents with the surface of smithsonite [22,24,28,32]. During the sulfidization process, ammonia or ammonium ions create an ionic adsorption layer on the mineral surface, leading to enhanced distribution and stability of the formed sulfide layer. The formation of these sulfidic products increases the adsorption capacity of collectors, ensuring high recovery in zinc oxide flotation processes [25]. In addition, ammonia or ammonium salts possess pH-regulating capabilities that provide an environment conducive to both sulfidization reactions and mineral flotation. Therefore, modification with ammonia–ammonium compounds represents a comprehensive and effective strategy to enhance surface sulfidization.

However, despite the obvious benefits of ammonia or ammonium salts in improving surface sulfidization as well as the flotation process of zinc oxide minerals, their utilization also entails certain drawbacks and potential risks. Ammonia has a powerful, pungent odor, and elevated concentrations in the production environment can cause adverse working conditions. Ammonia and specific ammonium salts, particularly in high concentrations, have the potential to cause irritation and harm to the respiratory system, skin, and eyes. Excessive use and improper disposal of ammonia and ammonium salts, especially when discharged as wastewater into natural water bodies, can result in water pollution. These limitations have prompted an exploration for more environmentally friendly alternatives that prioritize safety.

Triethanolamine (TEA) is an environmentally friendly compound with low toxicity and irritation. Previous studies have demonstrated the efficacy of TEA as a complexing agent, particularly its ability to chelate heavy metals [33,34]. This suggests that TEA may generate additional active sites by forming zinc–TEA complexes, which have promising potential to replace ammonia with ammonium compounds in enhancing the sulfidization process of the smithsonite surface.

In this study, the potential of TEA to enhance surface sulfidization and flotation of smithsonite was evaluated through micro-flotation experiments as an alternative to ammonia–ammonium compounds. X-ray photoelectron spectroscopy (XPS) with surface scanning and etching capabilities, field emission scanning electron microscopy, energy spectroscopy (FESEM–EDS), and atomic force microscopy (AFM) analysis were employed to examine the effect of TEA on the properties, structure, and distribution of sulfidization products. Zeta potential measurements, along with ab initio molecular dynamics (AIMD) and classical molecular dynamics (MD) calculations, were performed to gain a deeper understanding of the mechanisms by which TEA modification improves surface sulfidization [35,36]. This research explores the utilization of TEA as an alternative approach, while laying the foundation for future studies on more sustainable and efficient methods for zinc extraction.

## 2. Results and Discussion

### 2.1. TEA Improves the Flotation Behavior of Smithsonite

**Figure 1:**
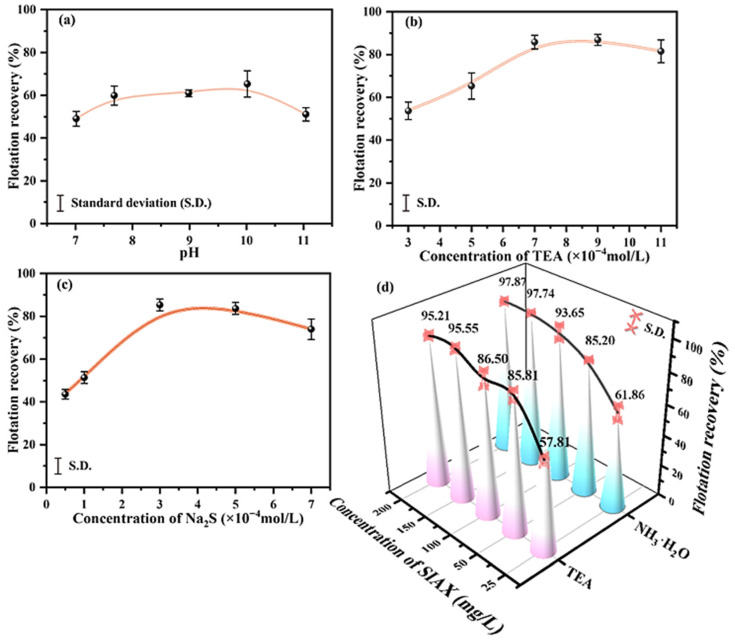
Smithsonite flotation recovery versus (**a**) pulp pH, (**b**) TEA concentration, (**c**) Na_2_S concentration, and (**d**) SIAX concentration under the following test conditions: (**a**) 5 × 10^−4^ mol/L TEA, 3 × 10^−4^ mol/L Na_2_S, 2 × 10^−4^ mol/L CuSO_4_, and 50 mg/L SIAX; (**b**) at pH around 10 at 3 × 10^−4^ mol/L Na_2_S, 2 × 10^−4^ mol/L CuSO_4_, and 50 mg/L SIAX; (**c**) at pH around 10 at 7 × 10^−4^ mol/L TEA, 2 × 10^−4^ mol/L CuSO_4_, and 50 mg/L SIAX; (**d**) at pH around 10, 3 × 10^−4^ mol/L Na_2_S, 2 × 10^−4^ mol/L CuSO_4_, surface modifier: 7 × 10^−4^ mol/L TEA or NH_3_·H_2_O.

The influence of pulp pH, TEA, and Na_2_S concentrations on smithsonite recovery was investigated, as shown in Figure 1. Smithsonite recovery increased with increasing pulp pH, with a peak of approximately 63% around pH 10, but then declined with a further increase in pH beyond 11 (Figure 1a). At a mildly alkaline pH around 10, HS^−^ significantly contributes to the sulfidization of smithsonite [37]. Recovery peaked at 81% with TEA concentrations increasing from 3 × 10^−4^ to 7 × 10^−4^ mol/L (Figure 1b), suggesting improved sulfide formation and product stabilization with TEA treatment. Smithsonite recovery increased to a peak of 83% when the Na_2_S concentration was increased to 3 × 10^−4^ mol/L (Figure 1c). However, a higher concentration of Na_2_S negatively affects smithsonite flotation.

Figure 1d shows the recovery of smithsonite modified with TEA or NH_3_·H_2_O as a function of SIAX concentration. In both modification cases, the recovery increased with the collector concentration. At 25 mg/L SIAX, the recoveries of TEA- and NH_3_·H_2_O-modified smithsonite were approximately 58% and 62%, respectively, while their recoveries reached a maximum of about 95% and 98% at 200 mg/L SIAX, respectively. These results demonstrate the significant improvement in smithsonite flotation recovery by TEA or NH_3_·H_2_O modification in a sulfidization xanthate flotation system. In addition, this highlights the potential of TEA as a cost-effective, environmentally friendly alternative to inorganic ammonium to improve surface smithsonite flotation in this system.

### 2.2. Contribution of TEA Modification to the Chemical State of Sulfidization Production

#### 2.2.1. Chemical State Characterization of Superficial Sulfidization Production

**Figure 2:**
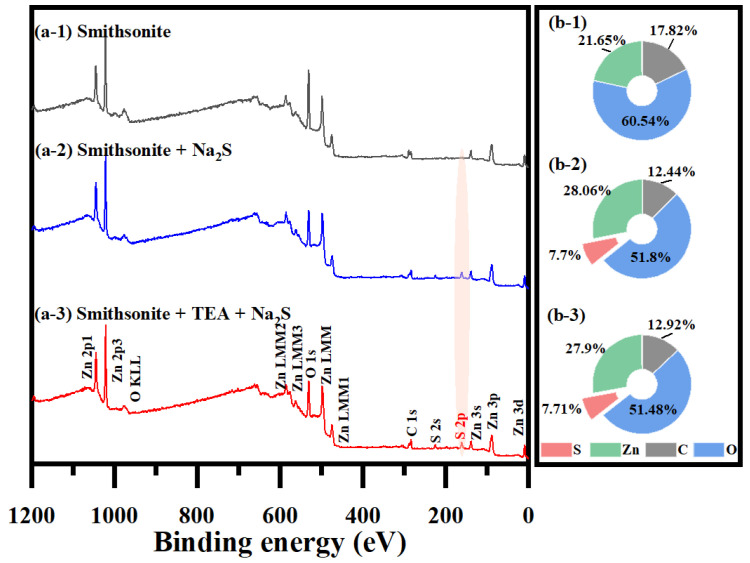
XPS: (**a**) full spectra and (**b**) relative atomic quantities of the surface of the smithsonite samples: without treatment, treated with Na_2_S, treated with TEA and Na_2_S.

**Figure 3:**
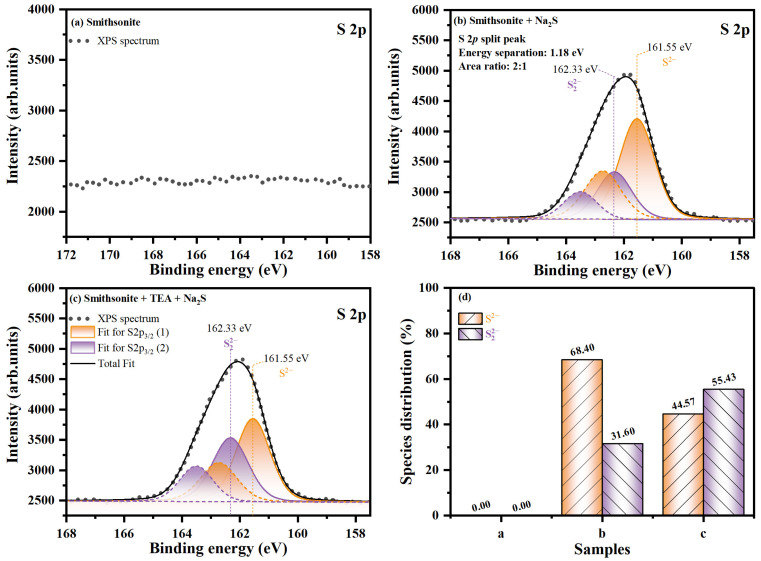
S 2p XP spectra on smithsonite sample surfaces: (**a**) without treatment, (**b**) treated with Na_2_S, and (**c**) treated with TEA and Na_2_S; and (**d**) PROP of different chemical states of S.

Elemental composition and chemical state of smithsonite surfaces were analyzed via XPS, with Figure 2 illustrates the full spectra and atomic ratios for Zn 2p_3/2_, C 1s (carbonate), O 1s, and S 2p [38,39]. The spectra show peaks for C, O, and Zn, but not for S in the untreated sample (Figure 2(a-1)). With or without TEA modification, sulfidization introduced S 2p peaks (Figure 2(a-2,a-3)). Na_2_S treatment alone increased surface S by 7.70% and Zn by 6.41% while decreasing C by 5.38% and O by 8.74%. Dual TEA and Na_2_S treatment raised S and Zn levels by 7.7% and 6.25%, respectively, and reduced C and O contents by 4.90% and 9.06%, respectively. These findings suggest that Zn carbonates and Zn hydroxyl groups may be converted to Zn sulfides during the sulfidization process.

**Figure 4:**
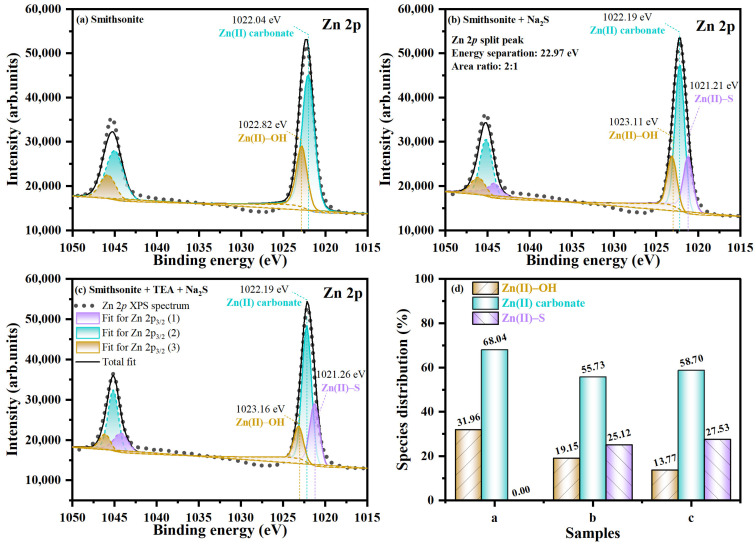
Zn 2p XP spectra on smithsonite sample surfaces: (**a**) without treatment, (**b**) treated with Na_2_S, and (**c**) treated with TEA and Na_2_S; and (**d**) PROP of different chemical states S.

Figure 3 and Figure 4 show the fine spectra of S 2p and Zn 2p, respectively, along with the distribution proportion of chemical states in the corresponding element, abbreviated as PROP (%). The fitting parameters are summarized in Table 1; the S 2p spectrum consists of S 2p_3/2_ and S 2p_1/2_ spin–orbit splitting peaks, with an energy separation of 1.18 eV and an area ratio of 2:1, as shown in Figure 3. In Figure 3a, no peaks are observed in the S 2p range on the surface of smithsonite without sulfidization. However, two doublets are present on the sulfidized smithsonite surface (Figure 3b,c), and their S 2p_3/2_ (1) and S 2p_3/2_ (2) peaks near 161.55 and 162.33 eV correspond to monosulfide (S^2−^) and disulfide (S_2_^2−^), respectively [27,40,41]. According to Figure 3d and Table 1, the PROP of S_2_^2−^ on the TEA-modified smithsonite surface significantly increased by 23.83% compared to the sample without TEA treatment, and its At increased by 1.01% within the range of X-ray detection. These results indicate that TEA modification induces the surface S^2−^ of the adsorbed smithsonite surface to lose electrons and be oxidized to S_2_^2−^, and S_2_^2−^ plays a significant role in improving the activity of sulfidization products and enhancing xanthate flotation [42].

The fitted Zn 2p spectra, with a composition of Zn 2p_3/2_ and Zn 2p_1/2_ peaks due to spin–orbit, are illustrated in Figure 4. On the untreated smithsonite surface, the Zn 2p spectrum comprised two doublets: one reveals a Zn 2p_3/2_ (2) peak showing a binding energy amounting to 1022.04 eV, and the second one manifests a Zn 2p_3/2_ (3) peak attributed to binding energy of 1022.82 eV (Figure 4a), which could be an outcome of the participation of Zn carbonate and hydroxide species, respectively [43,44,45,46,47]. The Zn 2p spectra of samples either treated with Na_2_S or with TEA and Na_2_S together (Figure 4b,c) are both composed of three doublets. The Zn 2p_3/2_(1) peak at 1021.25 eV was considered to be the Zn–S state on the smithsonite surface [40,48,49], while the binding energy near 1022.19 and 1023.15 eV for the Zn 2p_3/2_ (2) and Zn 2p_3/2_ (3) peaks, respectively, can be assigned to the Zn carbonates and hydroxides state, respectively. As presented in Figure 4d and Table 1, the PROP and At of the Zn–S state increased by 2.41% and 0.63%, respectively. Moreover, the presence of TEA modification significantly reduced the content of the Zn(II)–OH state on the sample surface, a change that could be due to the complexation of TEA with Zn hydroxyl species and Zn carbonates. The results suggest that complexation of TEA with Zn hydroxide and carbonate leads to the formation of additional Zn–TEA complex species that are chemisorbed onto the mineral surface, which results in an increase in the quantity and stability of Zn–S species during sulfidization.

#### 2.2.2. In-Depth Analysis of Sulfidization Production Chemical State

**Figure 5:**
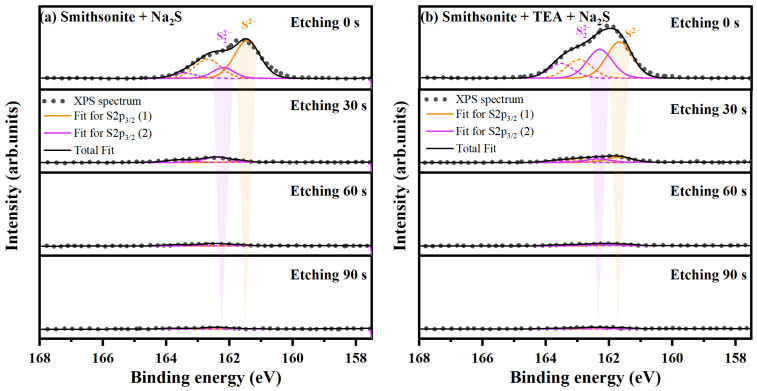
XP spectra of S 2p with different etching times for samples (**a**) treated with Na_2_S and (**b**) treated with TEA and Na_2_S.

The results of the XPS surface analysis indicate that the content of sulfidization products on the surface of smithsonite increases with the modification of TEA. However, the long-term stability of these sulfur species in the surface layer of smithsonite under various environmental conditions remains unclear [23,24]. Therefore, it is essential to investigate the influence of TEA modification on the quantity and chemical state of S species in the bulk phase of smithsonite through XPS depth profiling measurements and perform an etching test to gain insight into the composition and structure at different depths [50]. Figure 5 shows the narrow spectrum scanning of S 2p on sulfidized smithsonite from 0 to 90 s for argon ion etching, and the fitted parameters are presented in Table 2. The S 2p peaks consist of two doublet compositions, belonging to S^2−^ and S_2_^2−^, with binding energies in the range of 161.4 to 161.7 eV and 162.2 to 162.4 eV, respectively, in the absence and presence of TEA modification [27,40,41]. The intensity of the S 2p peak decreases significantly with increasing etching time, and the At of S^2−^ and S_2_^2−^ within both sample crystals decreases sharply. However, within the TEA-modified crystals, the At of S^2−^ and S_2_^2−^ is greater than that of S^2−^ and S_2_^2−^ within the crystals without TEA modification, and this difference diminishes with increasing sample depth. These results suggest that the modification of TEA can increase the content of S2 and S22 within the smithsonite crystals, and thus the stability of the sulfides within these crystals is higher than that of the surface sulfides.

### 2.3. Contribution of TEA Modification to the Distribution and Morphology of Sulfidization Production

**Figure 6:**
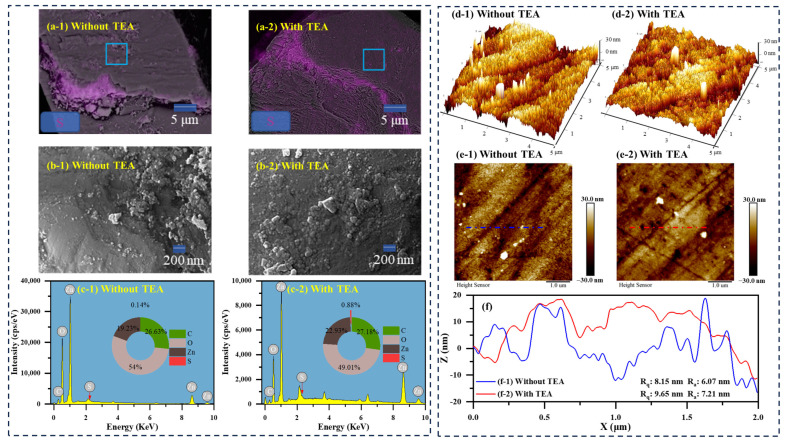
FESEM micrographs and EDS mappings (**a-1**–**b-2**, blue square: magnified region), EDS analysis results (**c-1**,**c-2**), AFM 3D and height images (**d-1**–**e-2**, blue and red dotted lines: AFM section), and height profiles (**f**) of smithsonite samples treated with Na_2_S, with and without the presence of TEA.

To thoroughly explore the effect of TEA modification on the distribution and morphology of the sulfidization products, both FESEM–EDS and AFM characterization techniques were employed, with the results shown in Figure 6 [51]. The FESEM–EDS probe analysis results indicate that after sulfidization treatment, there was a significant presence of S kα1 mapping on the smithsonite surface, particularly at the cracks. With the addition of TEA, the distribution of sulfidization products on the smithsonite surface increased, appearing as irregularly granular precipitates adhered to the surface. The sulfide compounds formed under TEA modification were larger and more evenly distributed, as seen in the comparison of the figures. This can be attributed to the TEA treatment causing the adsorption of Zn–TEA complexes onto the smithsonite surface, which can then form a stable and dense sulfur layer during sulfidization.

AFM analysis further examined the influence of TEA modification on the spatial size of particulate sulfide species on the smithsonite surface. Three-dimensional images and height maps indicate that sulfide products were widely distributed on the surface of the smithsonite, in particulate form, with or without TEA modification. The distribution of these sulfide products was more uniform, and their spatial size was more significant with TEA modification, as evidenced by the height curves. Characteristically, the presence of TEA modification resulted in a root mean square roughness (Rq) value of 9.65 nm and an average roughness (Ra) value of 7.21 nm, whereas in the absence of TEA modification, the Rq and Ra values were 8.15 nm and 6.07 nm, respectively. These results suggest that the TEA modification can increase the size of sulfide products. These enhancements are expected to improve the adsorption of activators and collectors onto the mineral, thereby improving the flotation recovery of smithsonite.

### 2.4. Effect of TEA Modification on the Smithsonite Surface Potential

**Figure 7:**
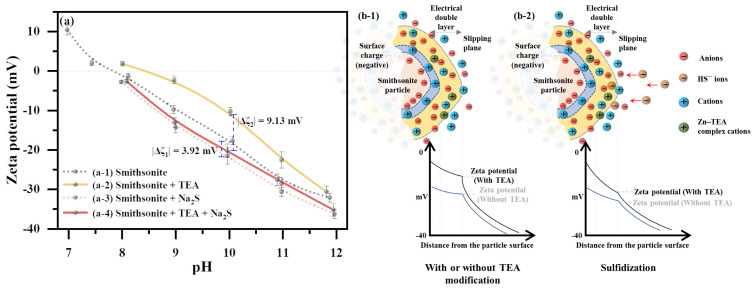
Effect of pH on zeta potential of smithsonite surface (**a**); electric double-layer models of smithsonite during TEA modification (**b-1**) and sulfidization (**b-2**).

The zeta potential (ζ-potential) of smithsonite surfaces under different conditions is shown in Figure 7a. The isoelectric point (pH_IEP_) of the unmodified sample was observed to be approximately pH 7.9, which is consistent with the literature [17,52]. Treatment with TEA alone significantly elevated the ζ-potential, shifting the pH_IEP_ to around 8.4. This rise is due to an increase in the cationic charge density within the Stern layer on the smithsonite surface, as reported in [53,54]. TEA forms neutral or cationic mono-, bi-, and polynuclear mixed ligand complexes with Zn ions on the surface, such as Zn(TEA)^2^⁺, Zn₂(TEA)_2_^4+^, and [Zn(TEA)]_n_^2n+^, depending on the pH and concentration conditions. The researchers for improved the concentration of Zn species in solution, including free Zn ions and Zn–bound TEA [55,56]. These Zn–TEA complex ions boost the charge density of cations within the Stern layer, thus raising the ζ-potential of the TEA-modified smithsonite (Figure 7(b-1)). Despite TEA surface modification, sulfidization resulted in a decline in the ζ-potential of the smithsonite surface (Figure 7a). Near the recommended pH of 10, the potential drop was more pronounced (9.13 mV) for surfaces treated with TEA compared to direct sulfidization (3.92 mV). The decrease indicates HS^−^ adsorption onto the mineral surface, given its dominance as a sulfide component around pH 10 [40]. However, the insignificant potential change in direct sulfidization confirms its limited impact on smithsonite (Figure 7(b-2)). The substantial difference in potential shifts on TEA-modified smithsonite highlights how TEA modification facilitates surface sulfidization. The presence of Zn–TEA complexes on the modified surface provides more active adsorption sites for sulfidizing agents and increases the surface cation density, thus increasing the likelihood of negatively charged HS^−^ adsorbing on mineral surfaces.

### 2.5. Mechanism of TEA Modification Enhancing Surface Sulfidization

#### 2.5.1. AIMD Analysis of TEA Contribution to Energy Change in Sulfidization Process

**Figure 8:**
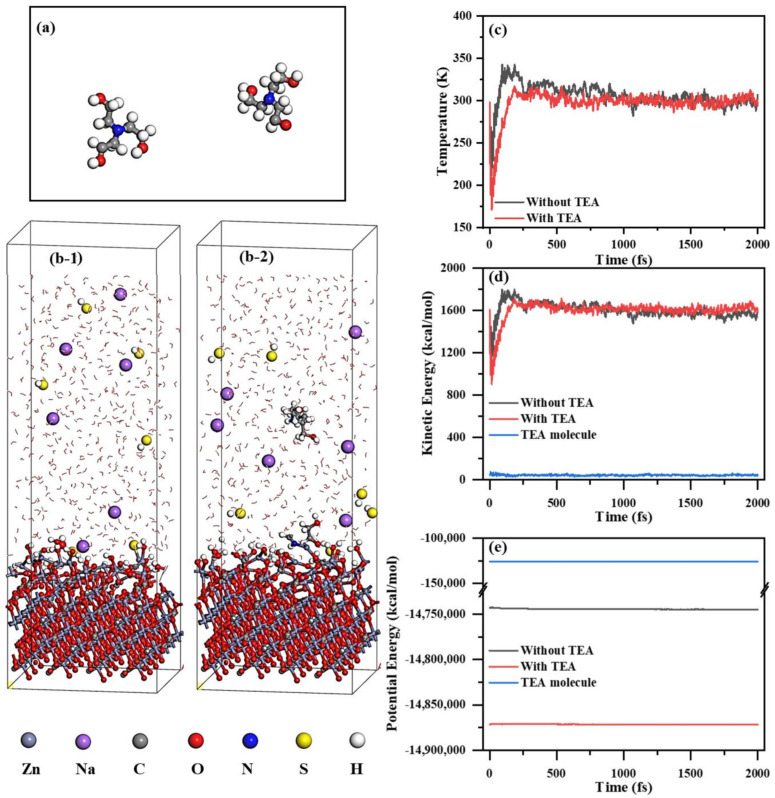
TEA molecules (**a**), adsorption models (**b-1**,**b-2**), temperature (**c**), kinetic energy (**d**), and potential energy (**e**) obtained from AIMD simulations.

Advanced AIMD simulation techniques were employed to elucidate the energy differences in the adsorption of NaHS onto the smithsonite (101) crystal plane under conditions with and without TEA (Figure 8a). The obtained data indicate that equilibrium is reached in the adsorption process after approximately 1000 fs, a conclusion supported by the simulation results depicted in Figure 8. Beyond 1000 fs, both the temperature evolution trend and energy change patterns (Figure 8c–e) exhibit clear signs of stabilization, thereby focusing the analysis on data beyond this time point. By comparing the visualized models at the 2000 fs timepoint, it is observed that HS^−^ interacts more closely with the smithsonite (101) crystal plane when TEA is present. This finding confirms that introducing TEA significantly enhances HS^−^ adsorption onto the smithsonite surface [57]. To quantify this enhancement effect, the intrinsic energy changes between systems with and without TEA were compared, including kinetic energy (Figure 8d) and potential energy (Figure 8e), as detailed in these figures. Analyzing intrinsic energy data from 1000 fs to 2000 fs reveals a significant reduction in energy within the system containing TEA during interaction with NaHS compared to that in the system without TEA, exhibiting an average intrinsic energy difference of –1465.69 kcal/mol. This substantial decrease in intrinsic energy provides compelling evidence for the positive promoting effect of TEA on sulfidization reaction processes. The modifying effect of TEA renders NaHS adsorption onto the smithsonite surface more stable and effective.

#### 2.5.2. MD Characterization of TEA Impact on the Adsorption Structure of Sulfide Layer

**Figure 9:**
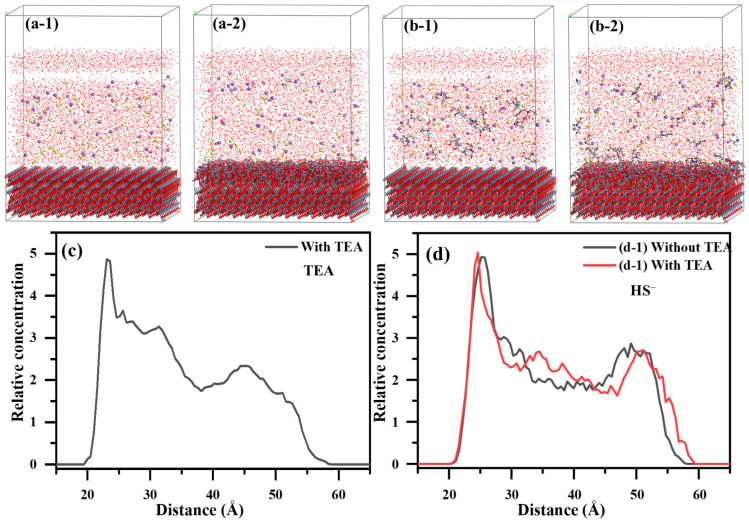
Initial (**a-1**,**b-1**) and equilibrium adsorption models (**a-2**,**b-2**) of the interactions between HS^−^ and the smithsonite (101) crystal plane in the absence and presence of TEA, along with the relative concentration distribution curves (**c**,**d**) of TEA and HS^−^ with respect to their vertical distances from the smithsonite (101) plane.

Based on classical MD simulations, the impacts of TEA on the molecular structure of adsorbed HS^−^ on the smithsonite (101) plane were thoroughly investigated. The simulation results demonstrate that the sulfide adsorption layers by the constituent HS^−^ were found on the mineral surface, regardless of the existence of TEA (Figure 9). However, with TEA present, it can adsorb onto the smithsonite surface and increase the quantity of closely adsorbed HS^−^ in the sulfide layer (Figure. 9(a-2,b-2)). This suggests that the introduction of TEA has a positive impact on the adsorption of HS^−^. The analysis of the concentration profile revealed distinct TEA adsorption layers located at distances of 23.07 Å and 31.37 Å from the surface (Figure 9c), corroborating the Zeta potential studies that show TEA can indeed adsorb onto the surfaces of smithsonite. The addition of TEA was found to effectively stabilize and densify the HS^−^ adsorption layer on the smithsonite surface. Moreover, while the HS^−^ adsorption layer peaks at 4.93 with a distance of 25.20 Å, it reaches a higher peak of 5.03 at a closer distance of 24.63 Å in the presence of TEA. These findings show that TEA promotes the adsorption of HS^−^ onto the smithsonite surface and enhances its stability (Figure 9d). Additionally, we observed a second TEA adsorption layer near the second adsorption layer of HS^−^ at a distance of 34.48 Å, further confirming that TEA strengthens the interaction between HS^−^ and the smithsonite surface. These simulation results demonstrate that TEA modification promotes closer interaction distances between HS^−^ and the smithsonite surface, resulting in a formation of multi-layer adsorption structures of HS^−^ on the smithsonite surface. It also reduces the energy required for the reaction between HS^−^ and the smithsonite surface, significantly enhancing the sulfidization process on the smithsonite surface.

**Figure 10:**
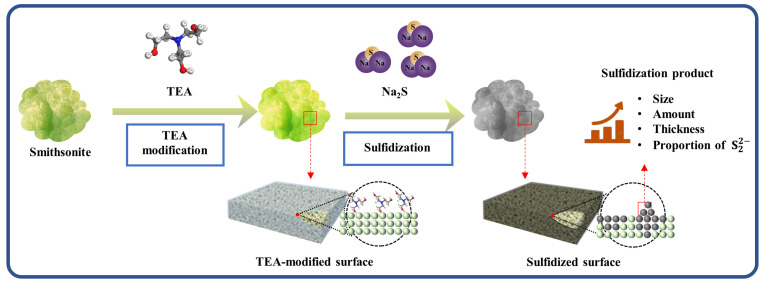
Schematic of TEA modification to enhance surface sulfidization of smithsonite.

Figure 10 shows a schematic diagram of the process of TEA modification to enhance the surface sulfidization of smithsonite. During the TEA modification process, Zn on the surface of smithsonite may form Zn–TEA complexes through coordination between TEA molecules and Zn ions. These complexes can chemically adsorb onto the mineral surface, and the positively charged complex ions increase the positive surface potential of the mineral. The adsorbed Zn–TEA complexes may serve as active adsorption sites for sulfidizing agents. The increase in positive surface potential in the mineral strengthens the probability that negatively charged HS^−^ is chemically adsorbed onto the surface. This enhancement mechanism is specifically demonstrated by the presence of TEA in the sulfidization process, which can reduce the total energy of the sulfidization reaction compared to that of the system without TEA. This enhancement effect of TEA results in an increased amount of sulfide compounds, an increased thickness of the sulfidization product layer, and an increase in the product size on the surface of sulfidized smithsonite. Furthermore, the proportion of advantageous S_2_^2−^ in the sulfidization product increases, enhancing its flotation properties.

## 3. Materials and Methods

### 3.1. Materials and Reagents

**Figure 11:**
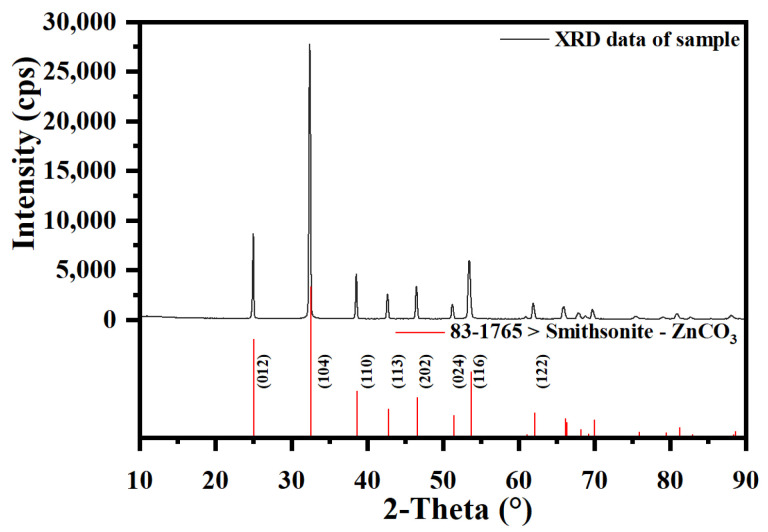
XRD pattern of smithsonite sample and reference JCPDS card.

The smithsonite specimens used in this study were obtained from Yunnan Province, China. The specimens were crushed, and the most prominent impurities were manually removed as thoroughly as possible. A portion of the crystals was ground to powder using a planetary mill and dry-sieved to yield two particle fractions measuring 38–74 μm and less than 38 μm, respectively. Another portion of the crystals was polished to prepare thin polished mineral sections. The diffraction pattern acquired from the X-ray of the ore sample resonated with the Joint Committee on Powder Diffraction Standards (JCPDS) database card (83–1765; Figure 11) [58], thus alluding to the high level of purity in the sample. To carry out the research, substances such as technically pure sodium isoamyl-xanthate (SIAX) and pine oil (Sourced from Tiefeng Mineral Processing Reagent, Kunming, China) were used in addition to the analytically pure form of hydrochloric acid (HCl), sodium hydroxide (NaOH), triethanolamine (TEA), ammonium hydroxide (NH_3_·H_2_O), sodium sulfide nonahydrate (Na_2_S·9H_2_O), and copper sulfate pentahydrate (CuSO_4_·5H_2_O). All experiments that were conducted used deionized water.

### 3.2. Experimental Methods

#### 3.2.1. Single Mineral Flotation

In a hanging laboratory flotation machine (XFGC_II_), the smithsonite particles (38–74 µm, 2.0 g) were added into 40.0 mL of water. The pH of the pulp was adjusted using dilute HCl and NaOH solutions. Subsequently, a prepared TEA or NH_3_·H_2_O solution was introduced and reacted for 5 min, followed by the addition of fresh Na_2_S solution for another 5-min reaction. The CuSO4 solution, the SIAX collector, and the pine oil frothing agent were individually added with 3 min, 2 min, and 1 min conditioning intervals. After the aeration, the foam was collected on the filter paper. The foam and residue were dried, weighed, and smithsonite recovery was calculated from the product’s mass. Each experiment was repeated 3 times for accuracy, with the results averaged and the standard deviation calculated.

#### 3.2.2. Characterization of Surface State and Morphology

For surface processing, powdered (–37 µm, 2.0 g) and smooth block (0.5 × 0.5 cm^2^) samples were used. These were combined with 0.5 L of deionized water in a reactor and treated with the reagent conditions of the optimal flotation result (TEA: 7 × 10^−4^ mol/L; Na_2_S: 3 × 10^−4^ mol/L, pH = 10 ± 0.1). The pulp was filtered; the residue was washed, dried under negative pressure (–0.08 MPa), and analyzed.

Both the particle and smooth block samples underwent XPS surface scanning and depth profiling. XPS data were collected with a K-Alpha^+^ instrument from Thermo Fisher Scientific, USA. The broad spectra scan utilized an Al Kα X-ray source (photon energy of 1486.68 eV) at an energy pass of 100 eV with a 1 eV step size. A pass energy of 50 eV with a 0.05 eV step size was used for the narrow spectrum. Depth profiling was achieved with Ar ion etching at a current of 10 nA, beam energy of 1000 eV, and a sputtering area of 0.01 × 0.01 cm^2^.

The surface morphology and energy spectrum of smithsonite were characterized using a high-resolution FESEM (CIQTEK, SEM 5000, Hefei, China) with an EDS. (OXFORD Xplore, Oxford, UK). Particle samples were mounted on electrically conductive tape; excess powder was removed and then gold-coated before analysis. The FESEM–EDS data were obtained under vacuum at 15 kV, 100 pA beam current, and a working distance of 12 mm. Additionally, smooth polished samples were scanned with an AFM (Dimension Icon, Bruker, Billerica, MA, USA) in tapping mode over a 5 μm × 5 μm area. AFM data analysis was performed with NanoScope Analysis 3.00 software. The AFM sample preparation involved cleaning the smithsonite surface with ethanol and deionized water, drying under nitrogen flow, and ensuring a smooth and contaminant-free surface. According to the instructions, the sample was treated with TEA and Na_2_S. After the treatment, the sample was dried with a nitrogen flow. Both height and phase images were obtained to identify surface features.

#### 3.2.3. Zeta Potential Measurement

Zeta potential measurement of the smithsonite particles was performed in a 1 × 10^−3^ M KCl electrolyte solution. Approximately 0.04 g of fine smithsonite (less than 5 μm) was added into 40.0 mL of the solution using a magnetic stirrer. After adding the TEA solution (7 × 10^−4^ mol/L), the suspension was conditioned for 5 min. Subsequently, the Na_2_S solution (3 × 10^−4^ mol/L) was introduced and allowed to react for another 5 min. The stirring prevented the particles from settling, forming a clear supernatant containing smithsonite, which was then extracted and transferred to an electrophoresis chamber using a pipette. The zeta potential was determined using a Nano-ZS90 instrument from Malvern Panalytical Ltd., Malvern, UK. Throughout the process, the pH value of the pulp was continuously maintained at the desired level. Each condition was measured 3 times, with the average recorded as the final data point.

#### 3.2.4. AIMD and MD Simulation

AIMD simulation was conducted using the CP2K package (version 2023.1), employing a combination of Gaussian and plane-wave methods with the Quickstep module [59]. The PBE exchange correlation functional was utilized, along with a molecularly optimized short-range Double-Zeta-Valence plus Polarization basis set featuring Goedecker–Teter–Hutter pseudo-potentials (DZVP-MOLOPT-SR-GTH). A plane-wave energy cutoff of 300 Ry was utilized, and Grimme’s dispersion correction with Becke–Johnson damping (D3BJ) was incorporated. Three-dimensional models of smithsonite, NaHS, TEA, and H_2_O were constructed, followed by geometry optimizations utilizing the LBFGS algorithm until convergence criteria for maximum step size, maximum number of geometry optimizations, and maximum geometry change in Ångstrom units converged to 0.2 Å and 500 iterations, respectively; additionally, the atomic units converged to 1.5 × 10^−3^ Å and 4.5 × 10^−4^ a.u, where NaHS is the hydrolysis product of Na_2_S. AIMD simulations of 6 NaHS, 2 TEA, and 500 H_2_O on the smithsonite slab model were performed under canonical ensemble conditions (NVT), running for a total of 2000 steps with a timestep set at 1.0 fs; thermostat-assisted velocity rescaling was employed as part of the canonical sampling process while initializing the system temperature at 298.15 K. Model construction and visualization tasks were facilitated by Materials Studio 2019 software. In addition, the input and output file processes were supported by the Multiwfn (version 3.8dev) package [60]. To quantify the promotion of TEA for the adsorption of NaHS onto the smithsonite surface, we calculated the difference in the interaction energy between NaHS and smithsonite in the presence and absence of TEA using Equations (1)–(3):

(1) $$\Delta E={(E}_{total1}-E_{TEA}^{0})-E_{total2}$$

(2) $$E_{total1}=E_{smithsonite}+E_{TEA}^{1}+E_{H_{2}O}+E_{sulfurizingreagent}$$

(3) $$E_{total2}=E_{smithsonite}+E_{H_{2}O}+E_{sulfurizingreagent}$$

E_total 1_ and E_total 2_ represent the intrinsic energy of smithsonite, H_2_O, and NaHS in equilibrium, with and without TEA, respectively. $E_{TEA}^{0}$ and $E_{TEA}^{1}$ represent the energies of isolated TEA and TEA in the system at equilibrium, respectively. A more negative ΔE indicates a more significant enhancement of the sulfidization on smithsonite due to TEA modification [61].

Classical MD simulation was performed using the Forcite module (Materials Studio 2019 software). Geometric optimizations for TEA, H_2_O, and NaHS were carried out using the B3LYP function within the DMol 3 module. The smithsonite (101) crystal plane was selected as the main focus of the investigation. An interaction model for the mineral–NaHS–H_2_O system was constructed using the Amorphous Cell module, incorporating TEA into the model according to research requirements. Under conditions involving TEA, the specific model consisted of 4000 H_2_O molecules, 15 TEA molecules, 40 Na^+^ cations, and 40 HS^−^ anions, supplemented by a protective layer of 1000 fixed H_2_O molecules. Prior to MD simulations, geometric optimization was applied to the entire model. Accurate charge was determined using the COMPASS II force field and charge equilibration method. The dynamic behavior of the NVT ensemble system was simulated at a constant temperature of 298 K, employing a Nose thermostat with a period of 500 picoseconds and a time step size of 1 femtosecond. Electrostatic interaction during the simulation was calculated using the Ewald summation method, and van der Waals interaction was computed utilizing an atom-based approach.

## 4. Conclusions

The present study investigated the potential of utilizing eco-friendly and cost-effective TEA as a substitute for traditional inorganic ammonia to optimize the flotation performance of smithsonite, while also providing an in-depth analysis of the mechanism by which TEA enhances the sulfidization process on the smithsonite surface.

Micro-flotation experiments showed a significant enhancement in flotation recovery of smithsonite with TEA modification. By introducing 7 × 10^−4^ mol/L TEA or aqueous ammonia under constant flotation conditions, the smithsonite recovery rates reached as much as 95.21% and 97.87%, respectively, indicating that TEA effectively replaces traditional inorganic ammonia and optimizes the sulfidization–xanthate flotation effect on smithsonite.

A comprehensive application of XPS, FESEM–EDS, and AFM were employed to elucidate the mechanism of the strengthening effect by TEA modification on the sulfidization process of smithsonite. The results revealed an increased content of sulfidization products on the surface of TEA-modified smithsonite, particularly a heightened proportion of crucial S_2_^2−^ in the adsorbed sulfides. Furthermore, there was an augmentation in both the thickness and size of the sulfidization product layer, resulting in a uniform and dense distribution on the smithsonite surface that enhanced adsorption stability.

The double-layer model analysis combined with comprehensive MD and AIMD simulations unveiled the action model for TEA modification to enhance the surface sulfidization process in smithsonite. An interaction between TEA and Zn sites on the smithsonite surface was observed as TEA coordinated to zinc atoms, forming positively charged Zn–TEA complexes which adsorbed onto the mineral surface. These positively charged complexes facilitated closer proximity between negatively charged HS^−^ and the smithsonite surface, leading to the formation of a multi-layered structure on its surface. Moreover, TEA reduced the energy requirements for the reaction between HS^−^ and smithsonite surfaces, significantly facilitating the sulfidization processes occurring at these interfaces. These findings reveal the intrinsic mechanism by which TEA modification enhances the flotation efficiency of smithsonite and offer novel insights for further optimizing flotation processes.

## Figures and Tables

**Table 1:** Distribution of elements on the sample surfaces: (a) without treatment, (b) treated with Na_2_S, and (c) treated with TEA and Na_2_S.

Elements	Samples	Core Peaks	BE./eV	FW. ^(I)^/eV	At ^(II)^/%	State
S	a	/	/	/	/	/
	b	S 2p_3/2_ (1)	161.55	1.41	5.27	S^2−^
		S 2p_3/2_ (2)	162.33	1.38	2.43	S_2_^2−^
	c	S 2p_3/2_ (1)	161.55	1.38	4.27	S^2−^
		S2p_3/2_ (2)	162.33	1.45	3.44	S_2_^2−^
Zn	a	Zn 2p_3/2_ (1)	1022.04	1.75	15.56	ZnCO_3_
		Zn 2p_3/2_ (2)	1022.82	1.52	6.09	Zn–OH
	b	Zn 2p_3/2_ (1)	1021.21	1.33	7.05	Zn–S
		Zn 2p_3/2_ (2)	1022.19	1.32	15.64	ZnCO_3_
		Zn 2p_3/2_ (3)	1023.11	1.33	5.37	Zn–OH
	c	Zn 2p_3/2_ (1)	1021.26	1.35	7.68	Zn–S
		Zn 2p_3/2_ (2)	1022.19	1.31	16.38	ZnCO_3_
		Zn 2p_3/2_ (3)	1023.16	1.29	3.84	Zn–OH

^(I)^ FW: full width at half maximum; ^(II)^ At: the atomic concentration of a chemical state within the specified range for testing.

**Table 2:** Distribution of elements on sample surfaces.

Etching Time/s	Sample	Smithsonite + Na_2_S		Smithsonite + TEA + Na_2_S	
		S^2−^	S_2_^2−^	S^2−^	S_2_^2−^
0	BE./eV	161.44	162.20	161.68	162.30
	FW./eV	0.96	0.81	0.96	0.94
	At/%	11.04	2.66	10.97	8.53
30	BE./eV	161.61	162.40	161.66	162.36
	FW./eV	0.60	0.96	0.96	0.96
	At/%	0.13	0.86	0.93	0.61
60	BE./eV	161.60	162.40	161.59	162.32
	FW./eV	0.61	0.96	0.96	0.96
	At/%	0.07	0.38	0.27	0.26
	BE./eV	161.40	162.40	161.60	162.40
	FW./eV	0.96	0.96	0.96	0.96
	At/%	0.03	0.26	0.17	0.21

## Data Availability

Data are contained within the article.
